# The importance of reaming the posterior femoral cortex before inserting lengthening nails and calculation of the amount of reaming

**DOI:** 10.1186/s13018-016-0345-6

**Published:** 2016-01-16

**Authors:** Metin Kucukkaya, Özgür Karakoyun, Mehmet Fatih Erol

**Affiliations:** Department of Orthopedics and Traumatology, Istanbul Bilim University, Büyükdere Cad. No:120 34394, Esentepe Sisli/Istanbul, Turkey; Department of Orthopedics and Traumatology, Namık Kemal University, Namık Kemal Üniversitesi Tıp Fakültesi Dekanlığı Namık Kemal Mahallesi Kampüs Caddesi No:1, Suleymanpasa/Tekirdağ, Turkey

**Keywords:** Limb lengthening, Lengthening nails, Posterior femoral cortex, Rigid reamer, Reaming

## Abstract

**Background:**

Lengthening nails have been used to correct limb length discrepancy caused by different etiologies, as well as for post-traumatic reasons. Two important lengthening nail-related complications are damage to the distraction mechanism and femoral fractures around the nail tip. As a result of the curved anatomy of the femur, straight nails impinge on the anterior cortex. Therefore, proper reshaping of the medullary canal to accommodate straight lengthening nails is crucial for the prevention of this problem. Reaming the dense posterior cortex is important when aiming to insert a lengthening nail without incurring anterior cortex nail tip impingement-related complications. Posterior femoral cortex over-reaming is a solution to this situation.

**Methods:**

Sixty patients received lengthening nails during 2008–2013, (ISKD, Fitbone, Precice). Posterior cortex rigid-reaming technique was used successfully in 45 retrograde femoral lengthening cases. The preoperatively planned posterior cortex amount was reamed until the impingement was overcome during the operation under fluoroscopic control for each case. Since the preoperative determination of posterior cortex reaming amount is time consuming and operator dependent, we evaluated the X-rays of the patients with computer software and conventional paper-based measurements. The effect of reaming the posterior cortical wall on the inclination of the nail tip to the anterior femoral cortex was detected with measurements on the preoperative and postoperative lateral femoral X-rays by using the CorelDRAW® Graphic Suite X6 software package (Corel, Inc., Ottawa, Ontario, Canada) software. On the same software, X-rays and the posterior reaming amount were also calculated.

**Results:**

The mean age of the patients was 27 years (11–42), while the mean lengthening was 5.9 cm (2–14). The mean consolidation index was 1.05 (0.75–1.62), and the mean follow-up period was 31 months (range, 18–45 months). The mean distance of the osteotomy site to the intercondylar notch of the femur was 81.2 mm (±16.92). The mean displacement of the nail tip position was 15.42 mm (±4.77) on the measurements on the postoperative X-rays after nail insertion compared to the preoperative simulations on the templates. The mean posterior cortex reaming thickness was 3.68 mm (±1.02).

**Conclusions:**

We derived a formula that allows the required amount of optimal posterior cortex reaming to be determined. No impingement-related complications or nail damage were observed.

## Background

Treatment techniques for leg-length discrepancies and deformities caused by post-traumatic or other reasons have developed substantially over the past decade. These techniques have been improved to allow the application of fully implantable lengthening nails [1, 2]. Lengthening nails are now available with various distraction mechanisms, such as mechanical (the Intramedullary Skeletal Kinetic Distractor (ISKD); Orthofix, McKinney, TX, USA), motor-driven (Fitbone nail; Wittenstein Intens, Igersheim, Germany), and magnetic (PRECICE; Ellipse Technologies, Irvine, CA, USA). Regardless of the lengthening mechanism, these devices are either straight or have an acute proximal angle. The main objective of this study was to emphasize the importance of rigid posterior femoral cortex reaming to avoid complications when inserting straight lengthening nails.

**Fig. 1:**
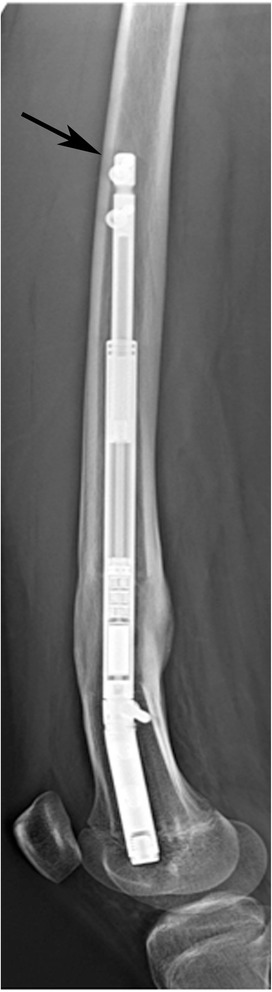
Lateral X-ray showing nail tip impingement on the anterior cortex in the proximal femur

Lengthening nails can be applied to the femur either antegradely or retrogradely [3]. In patients with additional distal femoral deformities, the simultaneous correction of acute deformities is also possible following distal femoral osteotomy in the retrograde technique [4]. In both applications, the tip of the straight nail impinges on the anterior cortex (Fig. 1). After the placement of locking screws, the durability of the anterior cortex decreases, which can result in fractures during either the operation or the lengthening period. Furthermore, the nail distraction mechanism itself can be damaged if the nails are hammered or forced during insertion into the bowed medullary canal.

**Fig. 2:**
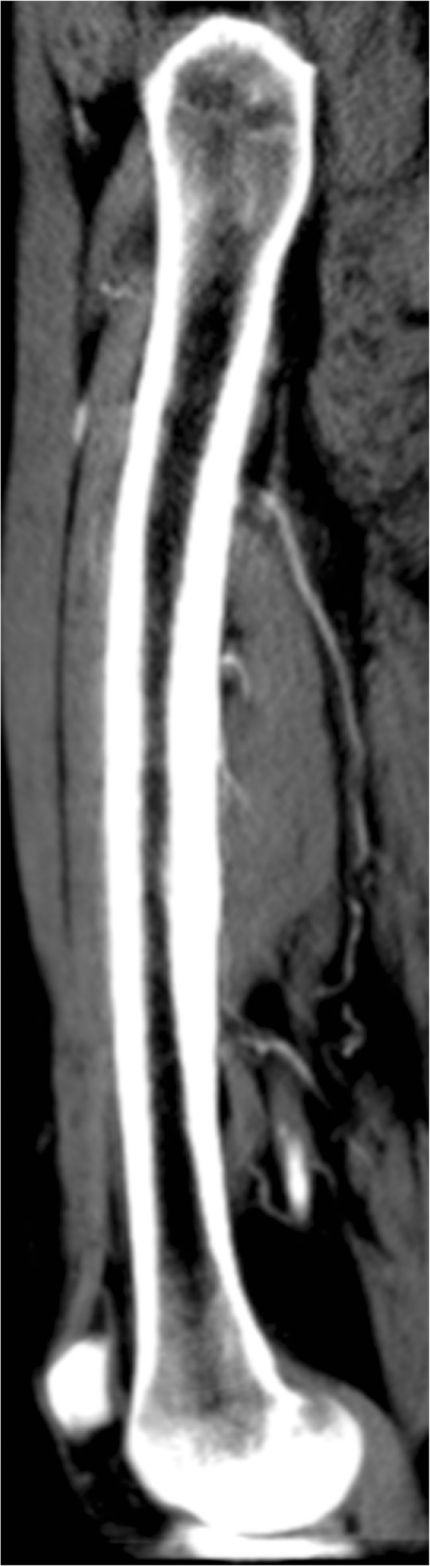
Computed tomography image of the femur in the sagittal section; the medullary canal has a greater antecurvature than that of the whole femur

A normal femur has a straight structure in the coronal plane. However, in the sagittal plane, it is bowed (antecurvature). The normal mechanical axis in a healthy individual passes posterior to the femur. As a result, the posterior femoral cortex is thicker in structure in the mid-diaphyseal region. Therefore, the posterior femoral cortex has a greater antecurvature than that of the whole femur (Fig. 2).

**Fig. 3:**
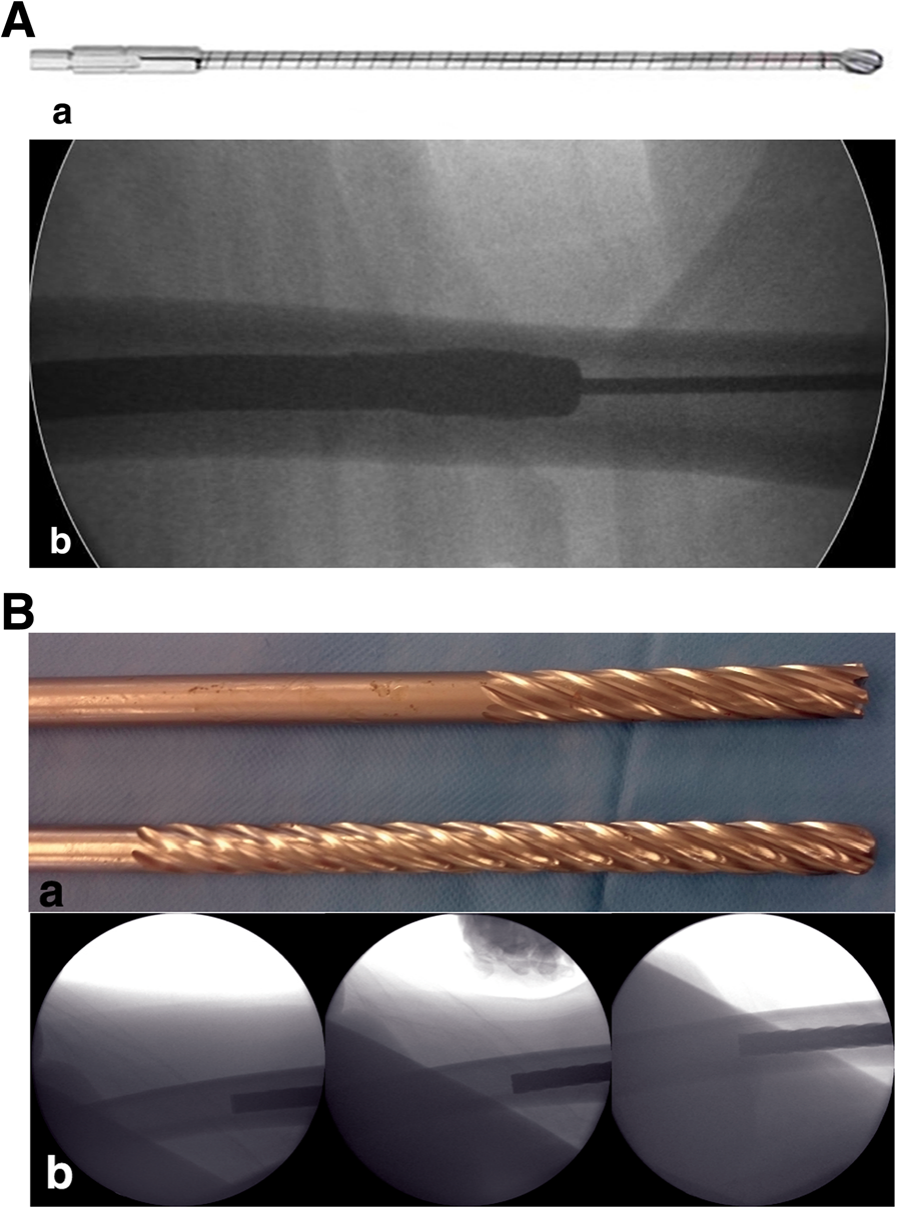
**a** Flexible reamer and fluoroscopic views of flexible reaming during the operation. **b** Rigid reamers; a sharp-edged reamer is used during the initial reaming for cutting the posterior cortex, while a round-shaped reamer is used for the smooth completion of the posterior reaming. Fluoroscopic views of rigid posterior cortex reaming during the operation

A widely used technique for the insertion of straight femoral lengthening nails is the flexible over-reaming of the medullary canal (Fig. 3a). However, this technique enlarges the canal rather than reshaping it. Over-reaming cannot completely protect the femur from anterior cortex nail tip impingement. In addition, it is not suitable for all femora, such as those with a very narrow medullary canal. The rigid-reaming technique includes the rigid reaming of the posterior cortex, which reshapes the medullary canal instead of enlarging it (Fig. 3b). With this technique, over-reaming is not necessary; it also provides a safe space in the proximal segment, avoids anterior cortex nail tip impingement, and facilitates the insertion of a straight nail.

We therefore (1) evaluated the effect of reaming the posterior cortical wall on the inclination of the nail tip to the anterior femoral cortex and (2) determined the optimal amount of posterior cortical reaming required with a rigid reamer using the CorelDRAW® Graphic Suite X6 software package (Corel, Inc., Ottawa, Ontario, Canada) and conventional paper-based measurements.

## Methods

Fifty-eight patients with 65 femoral shortenings of various etiologies underwent surgery with lengthening nails (ISKD, Fitbone, Precice). The etiologies of the femoral shortenings were malunion in 17 cases, physeal arrest in 12, poliomyelitis in 7, congenital hypoplasia in 8, achondroplasia in 1, and Legg-Calve-Perthes disease sequela in 2. There were five cases of idiopathic femoral shortenings and six cases underwent the procedure for cosmetic reasons. The cosmetic cases, and one of the achondroplasia cases, were treated for bilateral femurs. The retrograde insertion and rigid reaming techniques were used in 45 femora, and the antegrade insertion technique was used in 20 femora. The mean age of the patients was 27 years (range, 11–42 years), while the mean lengthening was 5.9 cm (range, 2–14 cm). The mean consolidation index was 1.05 (0.75–1.62), and the mean follow-up period was 31 months (range, 18–45 months). After the consolidation phases were complete, the patients were followed up at 3-month intervals during the first year. After the first year of consolidation, the follow-up was conducted yearly.

To detect the effect of reaming the posterior cortical wall on the inclination of the nail tip to the anterior femoral cortex in our cases, the preoperative and postoperative lateral femoral X-rays of the patients were uploaded to the Coral draw software. The template of the lengthening nail to be inserted was placed onto the preoperative X-rays without posterior cortex reaming so that the position of the nail tip without posterior cortex reaming and osteotomy was simulated. The distance of the nail tip to the posterior cortex was measured. Then, the distance of the nail tip to the posterior cortex was measured on the postoperative lateral femoral X-rays on which the nail tip was located in the intramedullary canal after posterior cortex reaming and angulation at the osteotomy site. By using these measurements, the displacement of the nail tip was calculated. The thickness of the thickest part of the posterior femoral cortex was measured on the preoperative and postoperative lateral femoral X-rays, and the posterior reaming amount was calculated.

### Rigid-reaming technique

**Fig. 4:**
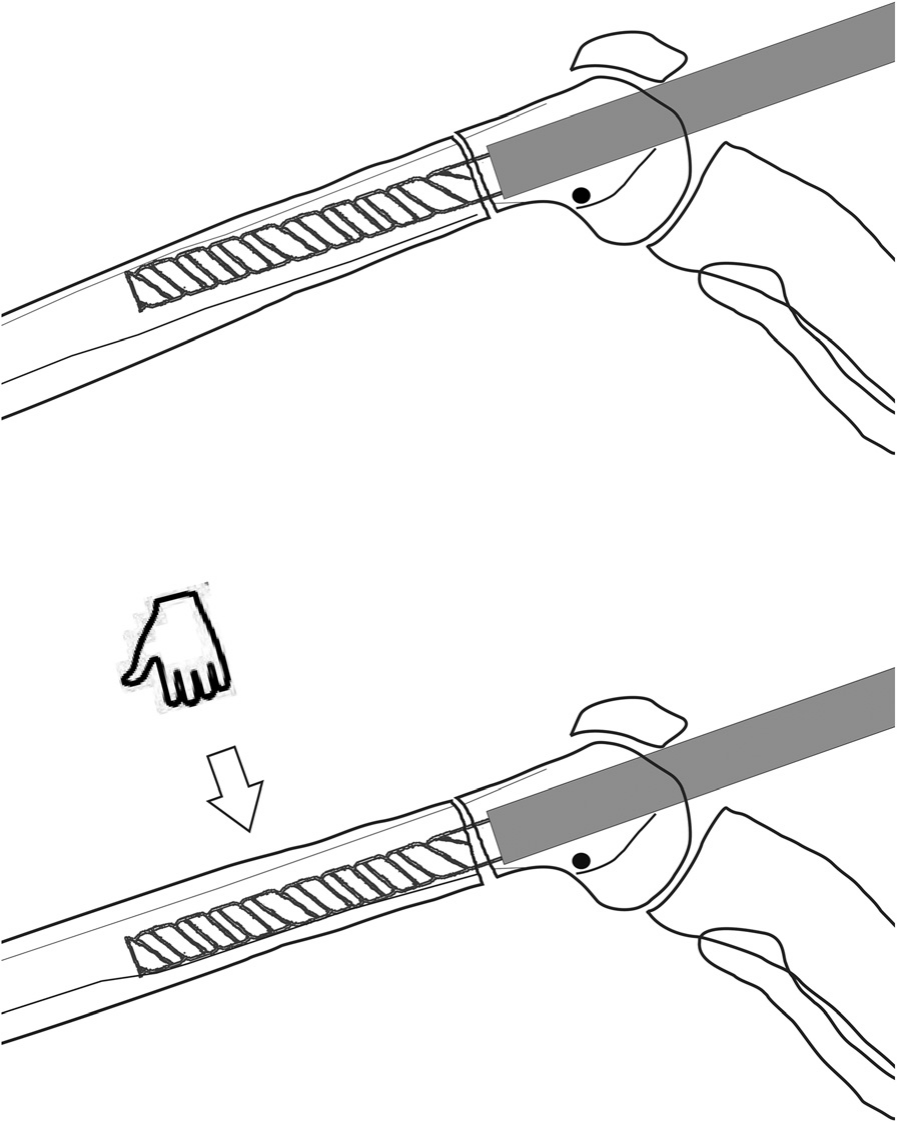
Preoperative planning of femoral lengthening using the CorelDRAW® Graphic Suite X6 software

In the retrograde technique, the patient was placed in the supine position on a radiolucent operating table with the knee in a semi-flexed position. In the antegrade technique, the patient was placed in the lateral decubitus position. A Kirschner (K)-wire was inserted at the standard entry point for retrograde femoral nailing on the medial part of the intercondylar notch in the anteroposterior view and at the apex of Blumensaat’s line, which is approximately 1 cm anterior to the posterior cruciate ligament in the lateral view. In the antegrade technique, a K-wire was inserted in the piriformis fossa for the straight lengthening nails. After the first entry, a rigid entry reamer was inserted over the K-wire to open the cortex, and the medullary canal of the proximal segment was reamed with a rigid reamer in all cases. Next, an osteotomy was created at the preoperatively planned site using the drill-osteotome method. Reaming of the dense posterior cortical wall of the distal segment was completed meticulously with a rigid reamer (Fig. 3b). This was necessary for the smooth and easy insertion of the lengthening nail and to prevent serious complications. The most important step in the procedure was the application of manual pressure onto the thigh from the anterior side to direct the rigid reamer posteriorly and to allow controlled and proper reaming (Fig. 4).

### Calculation of the amount of posterior cortex reaming required—“Software and conventional paper method”: sample case

**Fig. 5:**
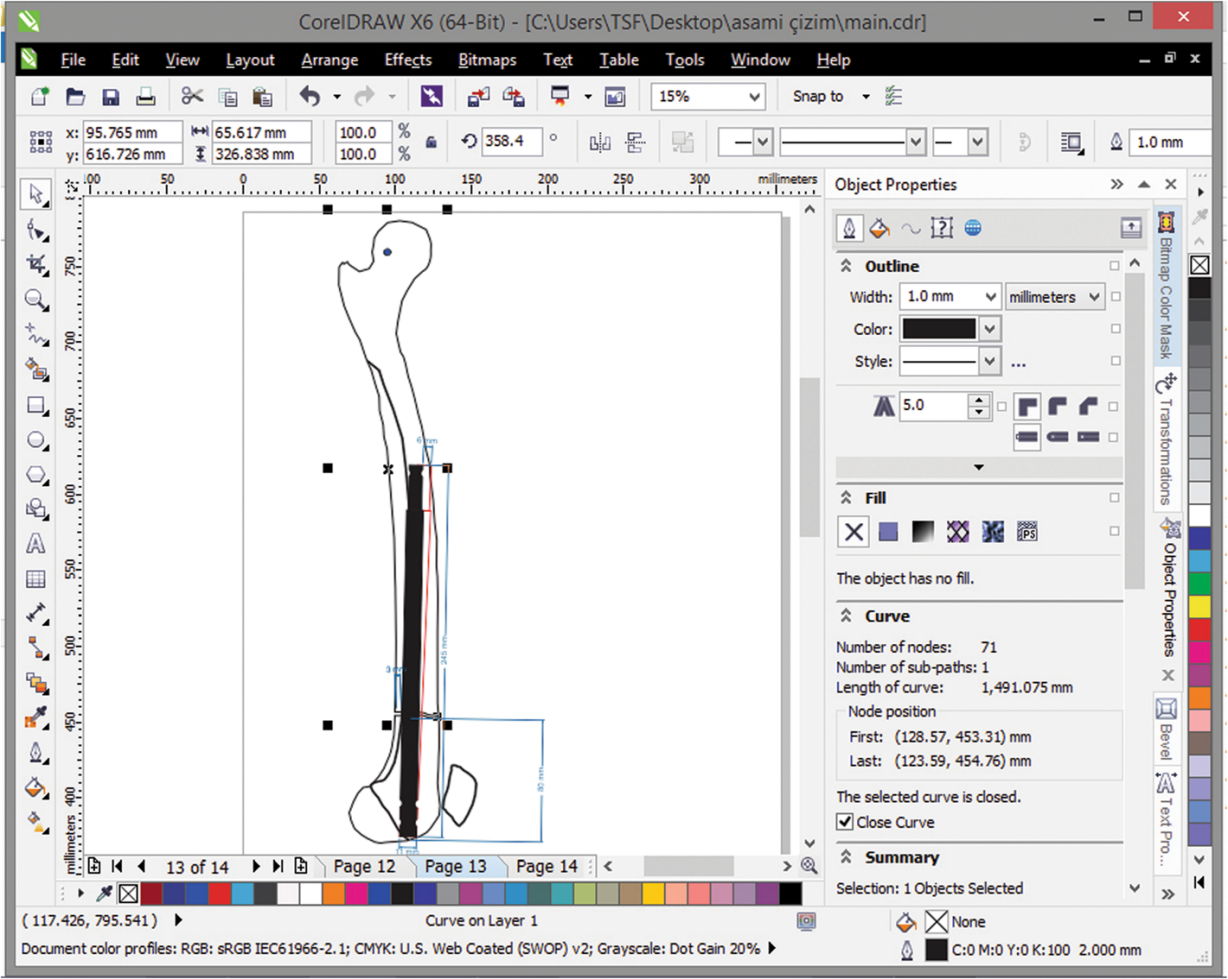
Illustration of manual pressure application onto the thigh from the anterior side, which is the most important step of the entire procedure and serves to direct the rigid reamer posteriorly and allow for controlled and proper reaming

Drawing paper and the CorelDRAW® Graphic Suite X6 software (Corel, Inc.) were used to evaluate the effect of reaming the posterior femoral cortex on the inclination of the nail tip to the anterior femoral cortex (Fig. 5).

**Fig. 6:**
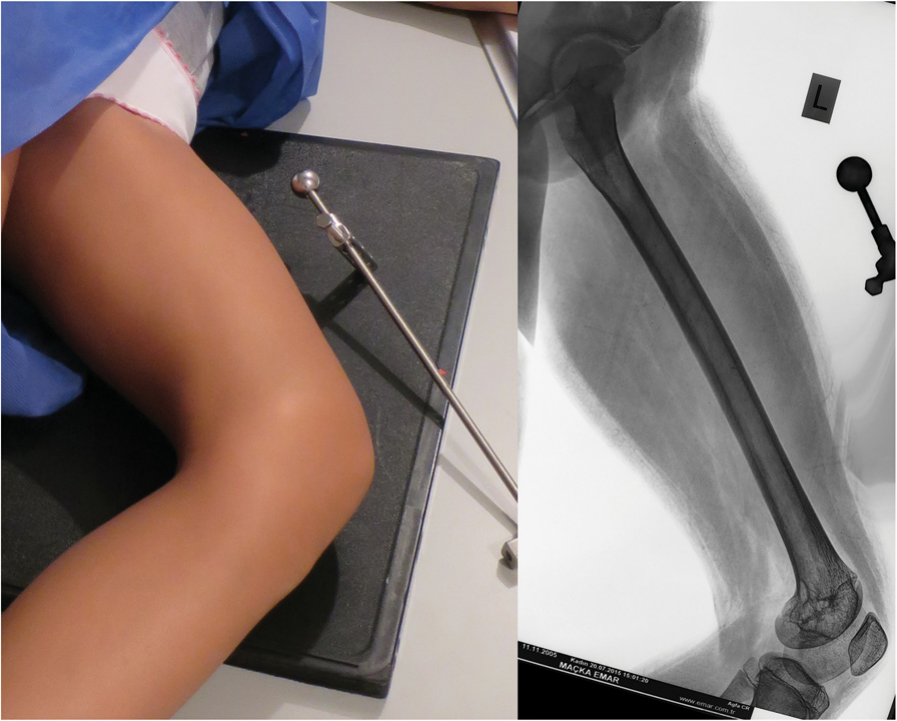
LAT X-ray of the femur. A 20-mm-diameter metallic sphere was positioned at the same distance from both the femur and the cassette for the calculation of the exact size of the femur

A lateral X-ray of a normal femur without any deformity or variation was obtained for the measurements and calculations. While the X-ray was being taken, a 20-mm-diameter metallic sphere was positioned at the same distance from both the femur and the cassette to allow the calculation of the exact size of the femur (Fig. 6). We uploaded the femoral radiogram to the software and also copied it onto the drawing paper. The anatomical lines and posterior cortex of the femoral radiogram were drawn using the software. Actual-size scaling was performed with the same software, using the known size of the metallic sphere. A virtual model of an 11-mm-diameter and 24.5-cm-long intramedullary nail (the most frequently used lengthening nail size) was produced. Nail protrusion was evaluated after unreamed and reamed retrograde application and after the osteotomy.

We then determined the amount of posterior cortical reaming required with a rigid reamer using the CorelDRAW® Graphic Suite X6 software (Corel, Inc.) and the conventional paper-based technique and derived a formula to allow the surgeon to predict the optimum amount of posterior cortex reaming required.

This study has been approved by “Istanbul Bilim University Clinical Researches Ethical Board” with the reference number 44140529/2014, and it is compliant with the Helsinki Declaration. An informed consent has been received from all participants or legal guardians for participation to the study.

## Results

The mean distance of the osteotomy site to the intercondylar notch of the femur was 81.2 mm (±16.92). The mean displacement of the nail tip position was 15.42 mm (±4.77) on the measurements on the postoperative X-rays after nail insertion compared to the preoperative simulations on the templates. The mean posterior cortex reaming thickness was 3.68 mm (±1.02).

### Sample case results

**Fig. 7:**
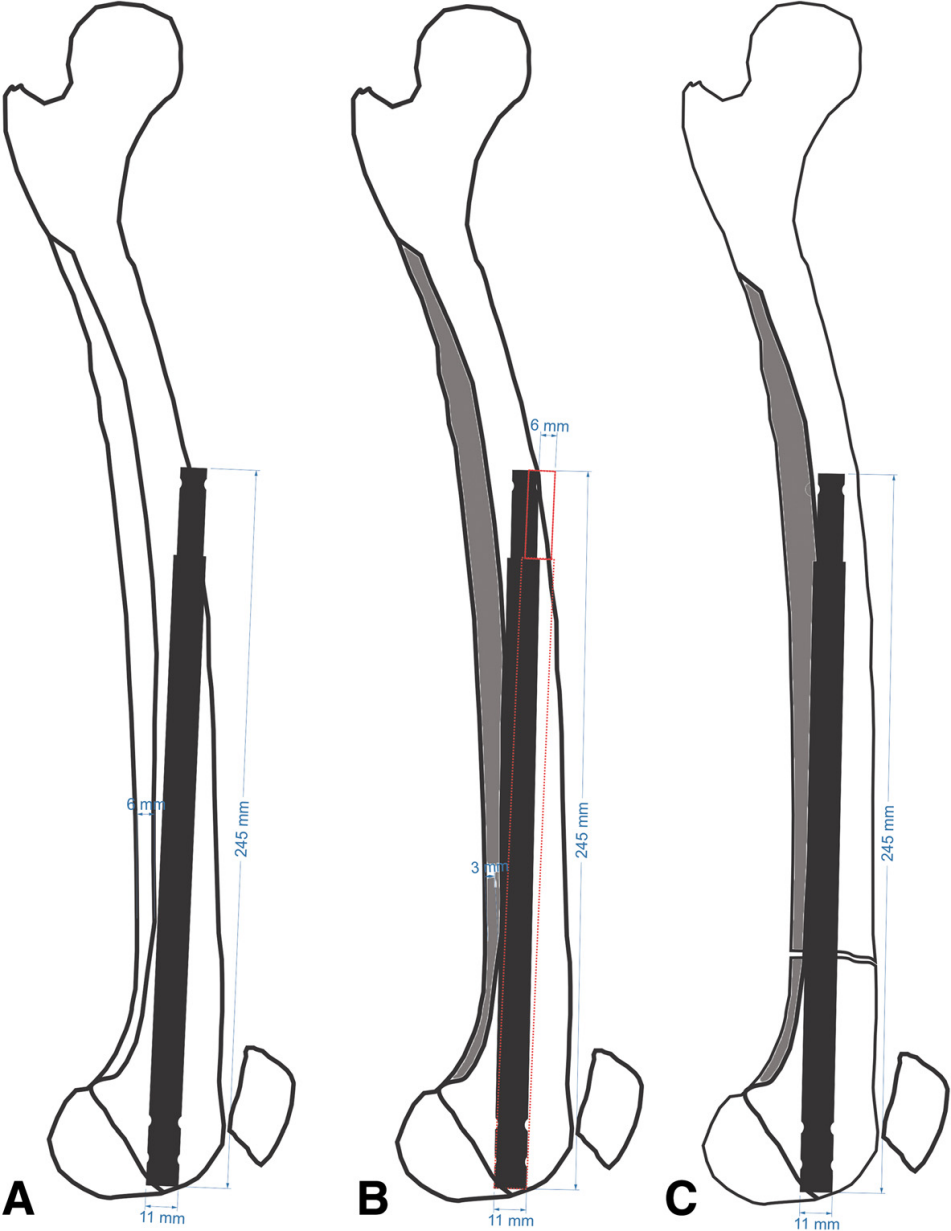
**a** A 6-mm protrusion of the nail tip on the anterior cortex in an unreamed application with a straight nail. **b** A 6-m posterior displacement of the nail tip after 3 mm of reaming 10 cm proximal to the intercondylar notch. **c** Greater than 6 mm posterior displacement of the nail in an osteotomized femur

**Fig. 8:**
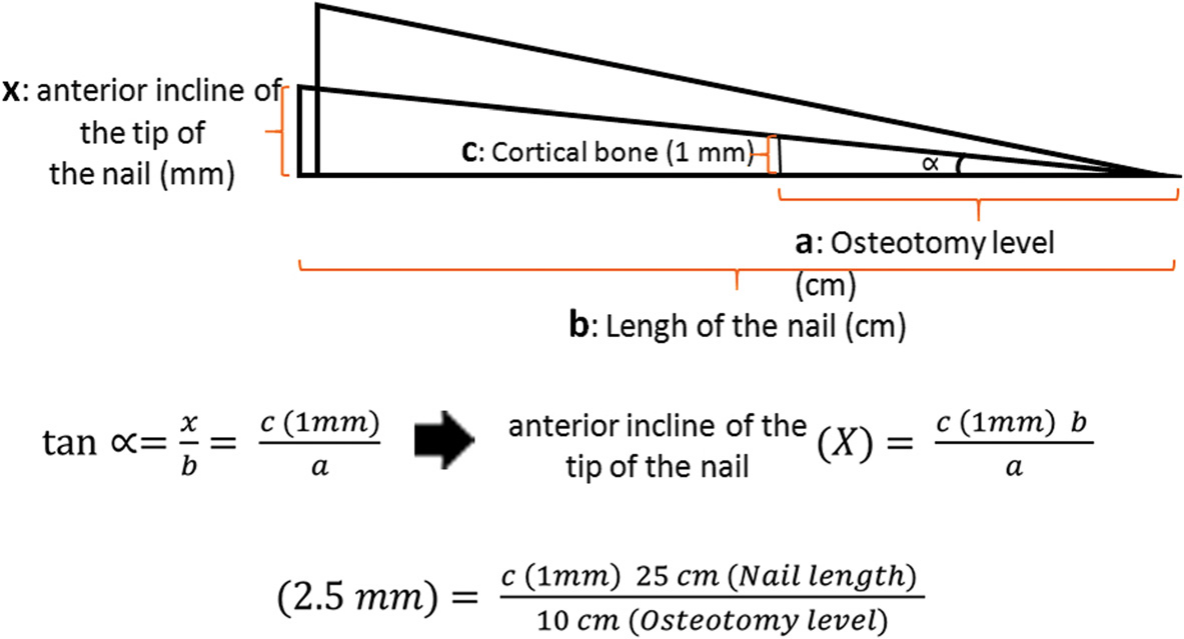
Formula for calculating the posterior displacement of a nail according to the required amount of reaming

The results of the measurements and calculations on the healthy femur revealed that the protruding part of the nail on the proximal anterior cortex was 6 mm in length, with an unreamed retrograde nail application (Fig. 7a). Posterior displacement of the nail tip was 6 mm when the posterior cortex was reamed by 3 mm at 10 cm proximal to the intercondylar notch (Fig. 7b). After the osteotomy, a recurvatum deformity occurred after the nail was inserted, contributing to the posterior movement of the nail tip, and the final nail tip posterior displacement was found to be >6 mm; this was sufficient for the prevention of the impingement (Fig. 7c). The tangent of the angle between the nail positions with and without posterior reaming is equal to the proportion of the anterior incline of the nail tip (*x*) to the nail length (*b*). It also equals the proportion of the reamed bone of the posterior cortex (*c*) to the distance to the osteotomy level from the intercondylar notch (*a*). After deriving our equation, we calculated the anterior incline in the nail tip according to the amount of posterior cortical reaming at a certain nail length and osteotomy level (Fig. 8).

## Discussion

Because the femur is bow-shaped in the sagittal plane, there are difficulties in adapting straight nails to the femoral anatomy. In femoral lengthening cases with both retrograde and antegrade lengthening nails, anterior nail tip impingement is a major problem that can cause femoral fractures or nail dysfunction. A straight lengthening nail can be inserted after reasonable over-reaming with a flexible reamer. However, posterior cortex rigid reaming, which solves the anterior nail tip impingement issue, is inevitable in cases with a narrow medullary canal or in patients who have an overcurved femur. The formula derived from our study may help the surgeon to easily predict the required amount of posterior cortex reaming. We experienced no anterior cortex impingement-related complications in our 65 femoral lengthening cases.

The anterior incline of the nail tip can be affected by sagittal bowing of the medullary canal or osteotomy level. The anterior incline of the nail tip decreases as the osteotomy level approximates the apex of the bow of the medullary canal. Buford et al. [5] built a cadaveric femoral model based on three-dimensional computed tomography. They calculated the mean sagittal radius of curvature (ROC) of the femur as 144.6 ± 39.7 cm. They also identified no difference in the ROCs between the anterior cortex and the medulla. Their study did not report the amount of posterior cortical bowing. However, the femur is more bowed in the posterior wall than in the anterior wall and medullary canal because the posterior femoral cortex has a greater thickness. Kanawati et al. [6] compared two intramedullary nails with ROCs of 150 and 200 cm after placement into synthetic femora. They reported that the amount of nail tip impingement on the anterior femoral cortex of the second nail was higher and recommended using nails with low ROCs to avoid anterior cortical impingement during applications. The lengthening nails are straight because at present the technology does not allow for the production of curved lengthening nails. Therefore, to produce a smooth insertion, it is crucial to adapt the shape of the femur to that of the straight nail.

The major limitation of this study was that we developed our formula using a healthy femur and a standard-sized nail. Therefore, the application of the formula to a femur with variations such as increased bowing or any other deformity in the coronal plane could cause technical difficulties such that the formula may need to be adjusted for each femoral variation.

## Conclusions

In conclusion, our formula is reliable in predicting the optimum amount of posterior cortex rigid reaming in femoral lengthening cases. The calculation of the amount of posterior cortex reaming during preoperative planning may not be necessary in all cases. However, preoperative planning should be carried out in all cases, especially in cases involving a narrow medullary canal, or patients with an overcurved femur. In addition, each patient should be evaluated separately in terms of any structural and histological variations. Our formula can be used as a mathematical proof of the planning. Our study can also open the way for developing more specific calculations for different clinical situations.
